# Supplementary material for: Inhibition of SRC-3 as a potential therapeutic strategy for aggressive mantle cell lymphoma
Source: PLoS One. 2024 Apr 29;19(4):e0289902. doi: 10.1371/journal.pone.0289902 (PMC11057735; doi:10.1371/journal.pone.0289902)
Supplement: Attachment — Submitted filename: Response to Reviewers.docx [file pone.0289902.s002.docx]

PONE-D-23-23808

Inhibition of SRC-3 as a potential therapeutic strategy for aggressive mantle cell lymphoma

Thank you for submitting your manuscript to PLOS ONE. After careful consideration, we feel that it has merit but does not fully meet PLOS ONE’s publication criteria as it currently stands. Therefore, we invite you to submit a revised version of the manuscript that addresses the points raised during the review process.

Please submit your revised manuscript by Jan 29 2024 11:59PM. If you will need more time than this to complete your revisions, please reply to this message or contact the journal office at plosone@plos.org. When you're ready to submit your revision, log on to https://www.editorialmanager.com/pone/ and select the 'Submissions Needing Revision' folder to locate your manuscript file.

Please include the following items when submitting your revised manuscript:

- A rebuttal letter that responds to each point raised by the academic editor and reviewer(s). You should upload this letter as a separate file labeled 'Response to Reviewers'.
- A marked-up copy of your manuscript that highlights changes made to the original version. You should upload this as a separate file labeled 'Revised Manuscript with Track Changes'.
- An unmarked version of your revised paper without tracked changes. You should upload this as a separate file labeled 'Manuscript'.

If you would like to make changes to your financial disclosure, please include your updated statement in your cover letter. Guidelines for resubmitting your figure files are available below the reviewer comments at the end of this letter.

If applicable, we recommend that you deposit your laboratory protocols in protocols.io to enhance the reproducibility of your results. Protocols.io assigns your protocol its own identifier (DOI) so that it can be cited independently in the future. For instructions see: https://journals.plos.org/plosone/s/submission-guidelines#loc-laboratory-protocols. Additionally, PLOS ONE offers an option for publishing peer-reviewed Lab Protocol articles, which describe protocols hosted on protocols.io. Read more information on sharing protocols at https://plos.org/protocols?utm_medium=editorial-email&utm_source=authorletters&utm_campaign=protocols.

We look forward to receiving your revised manuscript.

Kind regards,

Journal Requirements:

When submitting your revision, we need you to address these additional requirements.

1. Please ensure that your manuscript meets PLOS ONE's style requirements, including those for file naming. The PLOS ONE style templates can be found at

https://journals.plos.org/plosone/s/file?id=wjVg/PLOSOne_formatting_sample_main_body.pdf and

https://journals.plos.org/plosone/s/file?id=ba62/PLOSOne_formatting_sample_title_authors_affiliations.pdf

The manuscript has been updated for style based on links above

2. Thank you for stating the following in the Acknowledgments Section of your manuscript:

"The research was supported in part by National Institute of Health (R01-CA207701 to J.W., R01-CA250503 to J.W. and M.W.), Cancer Prevention & Research Institute of Texas (CPRIT, RP170500 to B.W.O. and J.W.), and the Michael E. DeBakey, M.D., Professorship in Pharmacology (to J.W.)."

We note that you have provided funding information that is not currently declared in your Funding Statement. However, funding information should not appear in the Acknowledgments section or other areas of your manuscript. We will only publish funding information present in the Funding Statement section of the online submission form.

Please remove any funding-related text from the manuscript and let us know how you would like to update your Funding Statement. Currently, your Funding Statement reads as follows:

"The research was supported in part by National Institute of Health (R01-CA207701 to J.W., R01-CA250503 to J.W. and M.W.), Cancer Prevention & Research Institute of Texas (CPRIT, RP170500 to B.W.O. and J.W.), and the Michael E. DeBakey, M.D., Professorship in Pharmacology (to J.W.)."

**Please include your amended statements within your cover letter; we will change the online submission form on your behalf.**

The funding information has been removed from manuscript

**3. We note that the grant information you provided in the ‘Funding Information’ and ‘Financial Disclosure’ sections do not match.**

When you resubmit, please ensure that you provide the correct grant numbers for the awards you received for your study in the ‘Funding Information’ section.

Here are the correct funding information: “The research was supported in part by National Institute of Health (R01-CA207701 to J.W., R01-CA250503 to J.W. and M.W.), Cancer Prevention & Research Institute of Texas (CPRIT, RP170500 to B.W.O. and J.W.), and the Michael E. DeBakey, M.D., Professorship in Pharmacology (to J.W.)."

**4. Thank you for stating the following financial disclosure:**

"The research was supported in part by National Institute of Health (R01-CA207701 to J.W., R01-CA250503 to J.W. and M.W.), Cancer Prevention & Research Institute of Texas (CPRIT, RP170500 to B.W.O. and J.W.), and the Michael E. DeBakey, M.D., Professorship in Pharmacology (to J.W.)."

Please state what role the funders took in the study. If the funders had no role, please state: "The funders had no role in study design, data collection and analysis, decision to publish, or preparation of the manuscript."

If this statement is not correct you must amend it as needed.

Please include this amended Role of Funder statement in your cover letter; we will change the online submission form on your behalf.

We added the following to the cover letter: "The funders had no role in study design, data collection and analysis, decision to publish, or preparation of the manuscript."

**5. Thank you for stating the following in the Competing Interests section:**

"J.W. is the co-founder of Chemical Biology Probes LLC. D.M.L. and B.W.O. are co-founders of CoRegen Inc. J.W. serves as a consultant for CoRegen Inc."

Please note Coactigon Inc has been dissolved in 2022. So it does not represent a conflict of interest and has been deleted from the statement.

We note that one or more of the authors are employed by a commercial company: CoActigon Inc. and Chemical Biology Probes, CoRegen Inc.

Jin Wang, David Lonard and Bert O’Malley are all employed by Baylor College of Medicine. Jin Wang founded Chemical Biology Probes LLC, whose business focus is irrelevant to this study. David Lonard and Bert O’Malley co-founded CoRegen Inc and their current research is partially supported by CoRegen Inc. Jin Wang currently serves as a paid consultant for CoRegen Inc. However,

a. Please provide an amended Funding Statement declaring this commercial affiliation, as well as a statement regarding the Role of Funders in your study. If the funding organization did not play a role in the study design, data collection and analysis, decision to publish, or preparation of the manuscript and only provided financial support in the form of authors' salaries and/or research materials, please review your statements relating to the author contributions, and ensure you have specifically and accurately indicated the role(s) that these authors had in your study. You can update author roles in the Author Contributions section of the online submission form.

Please also include the following statement within your amended Funding Statement.

“The funder provided support in the form of salaries for authors [insert relevant initials], but did not have any additional role in the study design, data collection and analysis, decision to publish, or preparation of the manuscript. The specific roles of these authors are articulated in the ‘author contributions’ section.”

If your commercial affiliation did play a role in your study, please state and explain this role within your updated Funding Statement.

The funder provided support in the form of salaries for authors [DML, BWO], but did not have any additional role in the study design, data collection and analysis, decision to publish, or preparation of the manuscript. The specific roles of these authors are articulated in the ‘author contributions’ section.

b. Please also provide an updated Competing Interests Statement declaring this commercial affiliation along with any other relevant declarations relating to employment, consultancy, patents, products in development, or marketed products, etc.

Within your Competing Interests Statement, please confirm that this commercial affiliation does not alter your adherence to all PLOS ONE policies on sharing data and materials by including the following statement: "This does not alter our adherence to PLOS ONE policies on sharing data and materials.” (as detailed online in our guide for authors http://journals.plos.org/plosone/s/competing-interests) . If this adherence statement is not accurate and there are restrictions on sharing of data and/or materials, please state these. Please note that we cannot proceed with consideration of your article until this information has been declared.

Please include both an updated Funding Statement and Competing Interests Statement in your cover letter. We will change the online submission form on your behalf.

Added.

6. In your Data Availability statement, you have not specified where the minimal data set underlying the results described in your manuscript can be found. PLOS defines a study's minimal data set as the underlying data used to reach the conclusions drawn in the manuscript and any additional data required to replicate the reported study findings in their entirety. All PLOS journals require that the minimal data set be made fully available. For more information about our data policy, please see http://journals.plos.org/plosone/s/data-availability.

"Upon re-submitting your revised manuscript, please upload your study’s minimal underlying data set as either Supporting Information files or to a stable, public repository and include the relevant URLs, DOIs, or accession numbers within your revised cover letter. For a list of acceptable repositories, please see http://journals.plos.org/plosone/s/data-availability#loc-recommended-repositories. Any potentially identifying patient information must be fully anonymized.

Important: If there are ethical or legal restrictions to sharing your data publicly, please explain these restrictions in detail. Please see our guidelines for more information on what we consider unacceptable restrictions to publicly sharing data: http://journals.plos.org/plosone/s/data-availability#loc-unacceptable-data-access-restrictions. Note that it is not acceptable for the authors to be the sole named individuals responsible for ensuring data access.

We will update your Data Availability statement to reflect the information you provide in your cover letter.

Excel sheet titled Dataset attached with data

7. Your ethics statement should only appear in the Methods section of your manuscript. If your ethics statement is written in any section besides the Methods, please move it to the Methods section and delete it from any other section. Please ensure that your ethics statement is included in your manuscript, as the ethics statement entered into the online submission form will not be published alongside your manuscript.

Ethics statement moved to methods section

8. PLOS ONE now requires that authors provide the original uncropped and unadjusted images underlying all blot or gel results reported in a submission’s figures or Supporting Information files. This policy and the journal’s other requirements for blot/gel reporting and figure preparation are described in detail at https://journals.plos.org/plosone/s/figures#loc-blot-and-gel-reporting-requirements and https://journals.plos.org/plosone/s/figures#loc-preparing-figures-from-image-files. When you submit your revised manuscript, please ensure that your figures adhere fully to these guidelines and provide the original underlying images for all blot or gel data reported in your submission. See the following link for instructions on providing the original image data: https://journals.plos.org/plosone/s/figures#loc-original-images-for-blots-and-gels.

In your cover letter, please note whether your blot/gel image data are in Supporting Information or posted at a public data repository, provide the repository URL if relevant, and provide specific details as to which raw blot/gel images, if any, are not available. Email us at plosone@plos.org if you have any questions.

Not applicable; no blots submitted with manuscript

9. Please review your reference list to ensure that it is complete and correct. If you have cited papers that have been retracted, please include the rationale for doing so in the manuscript text, or remove these references and replace them with relevant current references. Any changes to the reference list should be mentioned in the rebuttal letter that accompanies your revised manuscript. If you need to cite a retracted article, indicate the article’s retracted status in the References list and also include a citation and full reference for the retraction notice.

References 1 and 21 are corrected.

**Reviewers' comments:**

Question 1 cell model

The ibrutinib or venetoclex resistance is artificially produced. Do you know, or have hints if this resistance is similar to resistance seen in patients ? Did you test and/or find BTK mutations. ?

We (The Michael Wang group) previously reported that the resistance mechanism in Mino-venetoclax-R cells is associated with increased AKT phosphorylation and decreased PTEN levels (Lan et al., Clinical Cancer Research, 24(16), 2018). Currently, we are in the process of delineating the resistance mechanism for JeKo-R. Our observations reveal an upregulated OXPHOS pathway in JeKo-R cells (unpublished data), aligning with the observed ibrutinib resistance mechanism in patient samples (Sci Transl Med. 2019 May 8;11(491):eaau1167). We have not compared the resistance of the cell line to patient samples.

BTK mutation is a common drug resistant mechanism in chronic lymphocytic leukemia (CLL). However, in mantle cell lymphoma (MCL), BTK mutation is not a major resistant mechanism (Zhao et al. Unification of de novo and acquired ibrutinib resistance in mantle cell lymphoma. Nat Commun. 2017;8:14920.). Therefore, we did not test BTK mutations in this study. Based on DepMap.org database, Mino, Maver1, and Jeko1 have wild type BTK.

Question 2 long-term toxicty

Was there a concern for cardiotoxicity, as mentioned on page 2 ? You noted no weight changes in long-term treated mice.

Did you check for lab abnormalities troponin and pro-BNP, ?

We did not check troponin and pro-BNP. But we did look at the creatine kinase levels and did not observe statistical elevation of CK levels upon SI-10 treatments. The data has been added to the manuscript in Figure 5.

[NOTE: If reviewer comments were submitted as an attachment file, they will be attached to this email and accessible via the submission site. Please log into your account, locate the manuscript record, and check for the action link "View Attachments". If this link does not appear, there are no attachment files.]

While revising your submission, please upload your figure files to the Preflight Analysis and Conversion Engine (PACE) digital diagnostic tool, https://pacev2.apexcovantage.com/. PACE helps ensure that figures meet PLOS requirements. To use PACE, you must first register as a user. Registration is free. Then, login and navigate to the UPLOAD tab, where you will find detailed instructions on how to use the tool. If you encounter any issues or have any questions when using PACE, please email PLOS at figures@plos.org. Please note that Supporting Information files do not need this step.
